# Minisatellite mutation rates increase with extra-pair paternity among birds

**DOI:** 10.1186/1471-2148-9-100

**Published:** 2009-05-14

**Authors:** Anders P Møller, José J Cuervo

**Affiliations:** 1 Laboratoire d'Ecologie, Systématique et Evolution, CNRS UMR 8079, Université Paris-Sud, Bâtiment 362, F-91405 Orsay Cedex, France; 2 Estación Experimental de Zonas Áridas, CSIC, Calle General Segura 1, E-04001 Almería, Spain

## Abstract

**Background:**

Amos [1] suggested recently that a previously reported positive relationship between minisatellite mutation rates and extra-pair paternity among species of birds [2] was confounded by transcription errors and selective inclusion of studies. Here we attempted to replicate the results reported by Amos [1], but also tested for the relationship by expanding the data base by including studies published after our original paper.

**Results:**

We were able to replicate the positive association between mutation rate and extra-pair paternity in birds, even after controlling statistically for the confounding effecs of mean number of bands scored, using 133 species, compared to 81 species in our first report [2]. We suggest that Amos [1] failed to reach a similar conclusion due to four different potential causes of bias. First, Amos [1] missed 15 studies from the literature that we were able to include. Second, he used estimates of mutation rates that were based on both within- and extra-pair offspring, although the latter will cause bias in estimates. Third, he made a number of transcription errors from the original publications for extra-pair paternity, mutation rates, number of novel bands, and mean number of bands scored per individual. Fourth, he included *Vireo olivaceus *although the mutation rate estimate was based on one single offspring!

**Conclusion:**

There was a positive association between mutation rates and extra-pair paternity in birds, accounting for an intermediate effect size that explained 5–11% of the variance; estimates that are bound to be conservative due to many different causes of noise in the data. This result was robust to statistical control for potentially confounding variables, highlighting that it is important to base comparative studies on all available evidence, and that it is crucial to critically transcribe data while simultaneously checking published estimates for their correctness.

## Introduction

Our current understanding of the factors accounting for interspecific differences in mutation rates is at best poor [3]. We made a first attempt to address this lacuna by assembling a data base on mutation rates of minisatellites in different species of birds, produced as a result of a recent surge in studies investigating the evolution of extra-pair paternity [2]. The hypothesis tested was that sex differences in cell divisions could cause sex differences in mutation rates, and such sex differences should be more common in species with more intense sperm competition (see review of literature and justification for assumptions in [2]).

Amos [1] suggested that we made numerous transcription errors from the original sources, omitted key species, and selectively included species that supported our hypothesis. We contest these assertions strongly and later explain in detail how they arose. We also provide a comprehensive data base with all our data, and we show that our previous conclusion remains even when increasing sample size from 81 to 133 species due to recently published data that have become available after we finished our first study, and when controlling for a number of novel, potentially confounding variables.

## How to estimate mutation rates

We were puzzled to learn that Amos [1] had apparently not read or understood how we had estimated mutation rates, when we in fact described our procedures extensively [[2], p. 3, first column]. Given that Amos did not adopt the procedure that we used for estimating mutation rates, and given that he did not refute it on logical or other grounds, it is unsurprising that he finds extensive discrepancies between mutation rate estimates in the original papers and the rates that we reported. This does not justify claims about transcription errors or selective inclusion, but instead highlights the importance of reading papers before criticizing them.

Since Amos [1] apparently cannot understand our explicit descriptions [[2], p. 3], we have no other choice than repeating ourselves. Whenever possible we estimated mutation rates directly from data in the original publications by extracting information on the distribution of novel bands that could **not **be attributed to extra-pair paternity directly from the text or figures. We also extracted information on the total number of bands scored and the number of individuals used for these analyses. However, as we explicitly stated in Møller & Cuervo [[2], p. 3], we should not include all individuals in such estimates because offspring caused by extra-pair parentage ((male attending a nest is not the father) and extra-pair maternity (female attending a nest is not the mother) and intraspecific nest parasitism (neither male nor female attending a nest are parents)) will bias mutation rate estimates. We explicitly provided two examples in Møller & Cuervo [[2], p. 3], but we restrict this repeated explanation to the first of our examples. This first example concerns the indigo bunting *Passerina cyanea *for which Westneat [4] analyzed extra-pair paternity of 63 young of which 22 were extra-pair offspring. He found that 28 nestlings had 0 novel bands, 10 had 1 novel band, and 3 had 2 novel bands, in total 16 novel bands in 41 nestlings. Westneat [4] scored on average 37.5 bands or 41 young × 37.5 bands/young = 1537.5 bands in total. The mutation rate is therefore 16/1537.5 = 0.010407. However, many papers also included extra-pair offspring in their reported estimates of mutation rates. If we had done so, we would have had 63 young × 37.5 bands/young = 2362.5 bands in total. That would have given a mutation rate estimate of 16/2362.5 = 0.006772, or a reduction in mutation rate estimate by 35%. We justified clearly why extra-pair offspring should be excluded from such estimates because extra-pair offspring have many novel bands (in the example with the indigo bunting on average 8.2 novel bands). We stated explicitly in Møller & Cuervo [[2], p. 3] and we re-iterate here that "The latter precaution was taken since a single or a few novel band(s) in an individual due to mutation cannot readily be distinguished among a large number of novel bands due to extra-pair paternity". Thus 'hidden' mutational bands in extra-pair offspring will cause a bias in mutation rate estimates, and, therefore, they have to be excluded. Here we now report the frequency distribution of novel bands, the mean number of bands scored per individual and the number of individuals used for estimating mutation rates in Additional file 1, allowing readers to assess all the data and confirm our estimates.

Finally, we note that some of the estimates of mutation rates reported in the original publications were at conflict with what could be estimated using the number of novel bands, the mean number of bands scored and the number of individuals, and also in these cases have we used our own estimate rather than what was reported in the paper.

## Transcription of data

Amos [1] suggested that we made extensive transcription errors of mutation rates. We have already explained above why Amos [1] did not find consistency between our estimates and what was reported in the original publications. He also explicitly stated that the mutation rate estimate that we reported from Gibbs et al. [5] could not be found in the paper. Opening the pdf file of Gibbs et al. [5] with Acrobat reader allows anybody to search for 'mutation' and find the estimate of 0.018 on p. 368!

We do not have access to Amos' complete data set, because he chose not to publish it, although we assume that this is the data in his Additional file 1 combined with the data that we reported as corrected by him, but adjusted for what he terms 'our transcription errors'. However, we cannot know if that is the case because this is never stated explicitly. We have copied the data listed in Additional file 1 in Amos [1] and compared these values with what is reported in the original publications from which these data are claimed to have been extracted. We have found numerous errors. These range from the more mundane spelling errors in 9% of the species names (*Actitis hypoleucos*, *Carduelis tristis*, *Panurus biarmicus*, *Thryothorus ludovicianus*) to a difference in sample size of almost 1,000 for *Anthus spinoletta*. In addition, there are numerous errors in the frequency of extra-pair paternity, mutation rate, sample size and number of bands scored as reported in his Additional file 1. We report his data together with the data from the original publications in our Additional file 1 to allow readers to visualize these discrepancies. We suggest that someone who corrects others should be particularly careful not to make errors himself.

## Data selection criteria

Meta-analyses are strongly influenced by the data sets on which they are based, and it is always good scientific practice to report the data selection criteria adopted, but also to use multiple sources for accessing all available data [6-8]. We used all estimates of minisatellite mutation rates known to us in our first publication [2], and we would like to emphasize that one of us (APM) has kept an extensive list of all published studies of extra-pair paternity in birds since 1988, used for extensive analyses of the function of sperm competition [9,10]. We have never deliberately excluded any data, nor did we exclude *Vireo olivaceus*, contrary to what was suggested by Amos [1]. Close scrutiny of the original publication [11] for this species revealed that although the estimate of extra-pair paternity was based on 19 nestlings, in fact only 8 nestlings had information on minisatellite bands for both parents. Among these 8 nestlings, no fewer than 7 were extra-pair offspring, leaving one single nestling for scoring mutational bands. Inclusion of an estimate of mutation based on a sample size of one should be clearly inadequate to anybody including Amos!

As a measure of the completeness of the entire data base we would like to emphasize that we have identified 18 publications that were not in our original data set nor in the data set reported by Amos [1]. These papers are listed in our Additional file 2. As a second measure of completeness, we have kept a record of 48 manuscripts on extra-pair paternity that APM has refereed. Only one of these remains unpublished to date, suggesting that there is very little scope for any effects of publication bias in the analyses, contrary to what is commonly the case in meta-analyses [6-8].

## New analyses

Here we re-analyze the relationship between mutation rate and extra-pair paternity using the previously described procedures from Møller & Cuervo [2] and an extensive data set based on 133 species. In addition, we include five potentially confounding variables in the analyses. First, estimates based on large sample sizes will be more reliable than estimates based on small sample sizes, because the variance in estimates for small samples is greater than for large samples. Such patterns of decrease in sample variance with increasing sample size are ubiquitous in meta-analyses [6-8], and that is the main reason for including sample size as a confounding factor in the analyses. Thus it is not surprising that the variance also decreases with sample size in the present data for both mutation rates and extra-pair paternity. However, there is no reason to expect, as did Amos [1], that estimates of extra-pair paternity and mutation rates will be inherently small and hence under-estimated at small sample sizes, because they will simply only be more variable. Hence, there is good reason to control for sampling effort. Second, the mean number of bands scored varied among studies, and a larger number of bands may suggest a greater level of precision and hence a greater probability of detecting novel bands. Third, as we have argued previously [2], and also in the present study, correction of mutation rate estimates for extra-pair paternity may reduce bias because novel bands due to mutation 'hidden' among bands due to extra-pair parentage will not contribute to estimates. Therefore, we included this variable as a factor (with studies where we extracted the information on mutation rate directly from the publication being scored as 0, and studies where we estimated mutation rate after exclusion of extra-pair parentage were scored as 1) in the analyses because we could not correct all mutation rate estimates due to missing values. Fourth, while we originally analyzed minisatellite mutations, Amos [1] also included other molecular markers in the analyses. Hence, we included a factor that coded markers as minisatellites or other markers. Fifth, as we have already emphasized [2], molecular labs may differ in their procedures causing systematic differences in estimates of mutation rates among studies, and Amos [2] also suggested that there was a lab effect on estimates. Thus we included molecular lab as a factor in the analyses. Data for all these variables are provided in our Additional file 2 to allow readers to replicate our results and make further analyses. If more than one mutation rate and extra-pair paternity estimate was available for a species, we used mean estimates weighted by sample size for the analyses.

**Figure 1:**
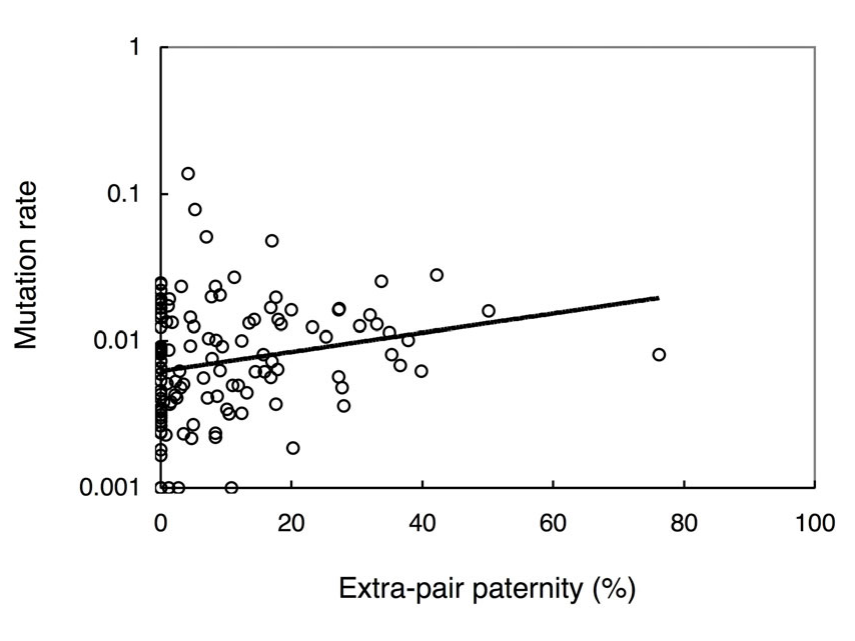
**Positive relationship between mutation rate and extra-pair paternity (% extra-pair young) in different species of birds**. Mutation rate was log_10_-transformed with a constant of 0.001 being added to avoid values of zero. The line is the linear regression line.

**Figure 2:**
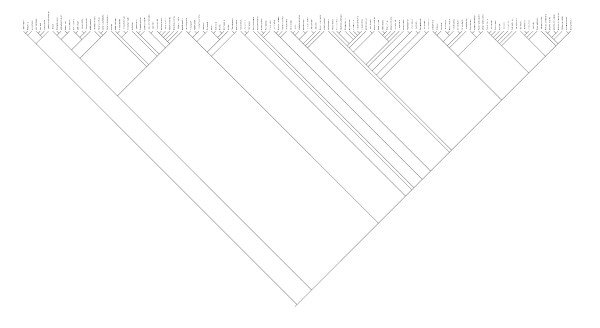
**Phylogenetic relationships between the species of birds included in the analyses**. Sources are listed in Møller & Cuervo [2], but now also include Hackett et al. [17].

The best-fit model relating extra-pair paternity to mutation rate including no potentially confounding variable explained 5.1% of the total variance (Table 1A). A model weighted by the square-root of sample size minus three (the standard error, [12]) did not provide a better fit (Table 1B). The relationship between mutation rate and extra-pair paternity is shown in Fig. 1. There was no significant additional effect of whether or not we extracted the mutation rate from the publication (*F *= 0.33, d.f. = 1,129, *P *= 0.57), whether the molecular marker was a minisatellite or not (*F *= 0.93, d.f. = 1,129, *P *= 0.34), or identity of the molecular lab (*F *= 1.05, d.f. = 50,80, *P *= 0.41). We constructed a phylogeny of all species (Fig. 2) for analyses of the relationship between mutation rate and extra-pair paternity, while simultaneously considering similarity in phenotype among species due to common phylogenetic descent. This phylogenetic analysis provided similar conclusions to the analysis based on species-specific data, explaining 8.6% of the variance (Table 1C). A phylogenetic analysis [12] weighted by the square-root of sample size minus three [13] explained 10.6% of the variance (Table 1D).

**Table 1:** Minisatellite mutation rates in different species of birds in relation to extra-pair paternity in (A) an analysis of species-specific unweighted data, (B) an analysis of species-specific weighted data, (C) a phylogenetic analysis of unweighted data, and (D) a phylogenetic analysis of weighted data.

Variable	Sum of squares	d.f.	*F*	*P*	Slope (SE)
**(A) Species-specific unweighted data**					
Extra-pair paternity	1.077	1	7.03	0.0090	0.397 (0.150)
Error	19.920	1			
					
**(B) Species-specific weighted data**					
Extra-pair paternity	9.242	1	6.87	0.0098	0.408 (0.156)
Error	174.815	1			
					
**(C) Phylogenetic analysis unweighted data**					
Extra-pair paternity	0.319	1	11.48	0.0009	0.524 (0.155)
Error	3.391	122			
					
**(D) Phylogenetic analysis weighted data**					
Extra-pair paternity	4.078	1	14.47	0.0001	0.611 (0.161)
Error	34.387	122			

Mutation rate was log_10_-transformed and a constant of 0.001 was added before transformation, extra-pair paternity was square-root arcsine-transformed.

## The relationship is conservative

The literature on mutation rates, and in particular the literature on mutations in minisatellites, is replete with comments on the difficulty of quantifying these, and Amos [1] cites a number of these references. We are the first to acknowledge these difficulties. However, we deliberately attempted to quantify the influence of any of these sources of error by calculating repeatabilities, using standard procedures from the quantitative genetics literature based on one-way analysis of variance [14]. In this way Møller & Cuervo [2] could show that despite large heterogeneity in estimates, there were still significant repeatability in mutation rate estimates and estimates of extra-pair paternity. We could also show that restriction enzyme as a factor (code 1 for a given study using an enzyme and 0 for all other studies) did not explain variation in mutation rate estimates [2]. We showed that the minimum size of fragments scored did not explain mutation rate estimates [2]. Finally, we showed that there was no significant effect of molecular lab [2], and there was no temporal improvement in techniques as shown by no significant effect of year of publication [2]. The absence of significant effects still holds in the currently much larger data set.

The main finding of our study is that mutation rates and extra-pair paternity are significantly positively related, and this relationship accounts for 5.1% of the variance in an analysis of species-specific data, and 8.6–10.6% of the variance in phylogenetic analyses, which equals a small to intermediate effect size (sensu Cohen [15], explaining 1% to 9% of the variance). We note that the effect size reported here is of the same magnitude that we originally reported (7.8%, [2]). This effect size is also very close to the average effect size in all meta-analyses in biology (around 5–7% of the variance explained [16]). Amos [1] emphasized all the difficulties in estimating mutation rates, and that any relationship will be conservative. We can only concur that the many sources of noise in the data will render any biological signal much weaker than the true underlying signal. Hence, we consider the effect size of 3.9% to be an underestimate.

## Conclusion

In conclusion, we have corroborated our previous conclusion [2] that mutation rates increase with extra-pair paternity in birds, with an effect size of small to intermediate magnitude. This conclusion is robust to inclusion of a number of potentially confounding variables and to statistical control for similarity in phenotype among species due to common phylogenetic descent.

## Authors' contributions

APM wrote the paper. APM and JJC extracted the data and finalized the paper. APM conceived of the study, and participated in its design and coordination. All authors read and approved the final manuscript.

## Supplementary Material

Additional file 1 **Supplementary table 1**. Information on species, research laboratory, number of individuals with 0, 1, 2, 3, 4, 5 or 6 novel bands due to mutations, mean number of bands scored (always from nestlings if there were data for both adults and nestlings), number of offspring used for mutation estimation, total number of offspring, estimated mutation rate, mutation rate as reported in the publication, extra-pair paternity (%; if there was information based on more than one probe, then the estimate based on the largest sample size), genetic marker (only that/those used for estimating mutation rates), whether the molecular marker was a minisatellite, whether the study was included by Amos, and reference. Click here for file

Additional file 2 **Supplementary table 2**. Comparative information on extra-pair paternity, mutation rate, sample size and mean number of bands scored as reported by Amos [1] and according to the publications. Click here for file
