# Supplementary material for: Minisatellite mutation rates increase with extra-pair paternity among birds
Source: BMC Evol Biol. 2009 May 14;9:100. doi: 10.1186/1471-2148-9-100 (PMC2693433; doi:10.1186/1471-2148-9-100)
Supplement: Additional file 1 — Supplementary table 1. Information on species, research laboratory, number of individuals with 0, 1, 2, 3, 4, 5 or 6 novel bands due to mutations, mean number of bands scored (always from nestlings if there were data for both adults and nestlings), number of offspring used for mutation estimation, total number of offspring, estimated mutation rate, mutation rate as reported in the publication, extra-pair paternity (%; if there was information based on more than one probe, then the estimate based on the largest sample size), genetic marker (only that/those used for estimating mutation rates), whether the molecular marker was a minisatellite, whether the study was included by Amos, and reference. [file 1471-2148-9-100-S1.doc]

Supplementary Table 1. Information on species, research laboratory, number of individuals with 0, 1, 2, 3, 4, 5 or 6 novel bands due to mutations, mean number of bands scored (always from nestlings if there were data for both adults and nestlings), number of offspring used for mutation estimation, total number of offspring, estimated mutation rate, mutation rate as reported in the publication, extra-pair paternity (%; if there was information based on more than one probe, then the estimate based on the largest sample size), genetic marker (only that/those used for estimating mutation rates), whether the molecular marker was a minisatellite, whether the study was included by Amos [1], and reference. References are listed in Møller & Cuervo [2] and Amos [1], and if not included there, the references are listed below.

| Species | Research laboratory | No. individuals with 0 novel bands | No. individuals with 1 novel band | No. individuals with 2 novel bands | No. individuals with 3 novel bands | No. individuals with 4 novel bands | No. individuals with 5 novel bands | No. individuals with 6 novel bands | Mean no. bands scored |
| --- | --- | --- | --- | --- | --- | --- | --- | --- | --- |
| *Acrocephalus arundinaceus* | 1 | 484 | 48 | 4 | 0 | 0 | 0 | 0 | 27.3 |
| *Acrocephalus melanopogon* | 22 | 13 | 5 | 0 | 0 | 0 | 0 | 0 | 17.9 |
| *Acrocephalus schoenobaenus* | 1 | 115 | 27 | 1 | 0 | 0 | 0 | 0 | 33 |
| *Acrocephalus schoenobaenus* | 34 | 81 | 28 | 7 | 1 | 0 | 0 | 0 | 61.1 |
| *Acrocephalus vaughani* | 2 | 20 | 0 | 0 | 0 | 0 | 0 | 0 | . |
| *Actitis hypoleucos* | 2 | 45 | 10 | 5 | 0 | 0 | 0 | 0 | 47.1 |
| *Agelaius phoeniceus* | 3 | 104 | 47 | 18 | 6 | 1 | 0 | 0 | 71.6 |
| *Agelaius phoeniceus* | 4 | . | . | . | . | . | . | . | 30.5 |
| *Alectura lathami* | 51 | 35 | 5 | 0 | 0 | 0 | 0 | 0 | 26.2 |
| *Anser caerulescens* | 9 | 74 | 0 | 1 | 0 | 0 | 0 | 0 | 15.7 |
| *Anser rossi* | 9 | 76 | 5 | 0 | 0 | 0 | 0 | 0 | 20 |
| *Anthus spinoletta* | 52 | 96 | 125 | 191 | 164 | 86 | 19 | 0 | 27.2 |
| *Aphelocoma coerulescens* | 35 | 110 | 27 | 2 | 0 | 0 | 0 | 0 | 40.5 |
| *Apus apus* | 5 | 62 | 14 | 4 | 0 | 0 | 0 | 0 | 19.4 |
| *Asio otus* | 8 | 56 | 3 | 0 | 0 | 0 | 0 | 0 | 21.5 |
| *Athene noctua* | 16 | 49 | 4 | 0 | 0 | 0 | 0 | 0 | 20.95 |
| *Branta leucopsis* | 51 | 8 | 5 | 1 | 0 | 0 | 0 | 0 | 25.8 |
| *Branta leucopsis* | 51 | 8 | 5 | 1 | 0 | 0 | 0 | 0 | 22.1 |
| *Branta leucopsis* | 6 | 112 | 2 | 0 | 0 | 0 | 0 | 0 | 21.4 |
| *Buteo galapagoensis* | 36 | 18 | 1 | 0 | 0 | 0 | 0 | 0 | 37.9 |
| *Calidris maritima* | 7 | 66 | 14 | 1 | 0 | 0 | 0 | 0 | 25.6 |
| *Calidris mauri* | 22 | 41 | 11 | 5 | 0 | 0 | 0 | 0 | 32 |
| *Calonectris diomedea* | 37 | 29 | 3 | 2 | 0 | 0 | 0 | 0 | 26.7 |
| *Campylorhynchus griseus* | 8 | 176 | 30 | 1 | 0 | 0 | 0 | 0 | 33 |
| *Campylorhynchus griseus* | 8 | . | . | . | . | . | . | . | . |
| *Campylorhynchus nuchalis* | 8 | 58 | 10 | 0 | 0 | 0 | 0 | 0 | 54.4 |
| *Cardinalis cardinalis* | 3 | 21 | 9 | 2 | 0 | 0 | 0 | 0 | 29.6 |
| *Cardinalis cardinalis* | 3 | 21 | 9 | 2 | 0 | 0 | 0 | 0 | 38.1 |
| *Carduelis tristis* | 53 | 42 | 10 | 2 | 0 | 0 | 0 | 0 | 20 |
| *Carpodacus mexicanus* | 9 | 71 | 2 | 0 | 0 | 0 | 0 | 0 | 22.5 |
| *Catharacta lonnbergi* | 52 | . | . | . | . | . | . | . | 29.3 |
| *Catharacta maccormicki* | 52 | 11 | 1 | 0 | 0 | 0 | 0 | 0 | 26.85 |
| *Cercomacra tyrannina* | 11 | 4 | 5 | 2 | 0 | 0 | 0 | 0 | 35 |
| *Charadrius alexandrinus* | 22 | 118 | 43 | 6 | 0 | 0 | 0 | 0 | 18 |
| *Charadrius hiaticula* | 22 | 38 | 9 | 3 | 0 | 0 | 0 | 0 | 17.9 |
| *Charadrius semipalmatus* | 12 | 60 | 2 | 0 | 0 | 0 | 0 | 0 | 27.3 |
| *Coragyps atratus* | 8 | 33 | 3 | 0 | 0 | 0 | 0 | 0 | 21.7 |
| *Coragyps atratus* | 8 | 32 | 3 | 1 | 0 | 0 | 0 | 0 | 22.8 |
| *Corcorax melanoramphos* | 19 | 37 | 12 | 2 | 0 | 0 | 0 | 0 | 39.9 |
| *Corvus monedula* | 2 | . | . | . | . | . | . | . | 15.41 |
| *Cyanoliseus patagonus* | 23 | 151 | 12 | 1 | 0 | 0 | 0 | 0 | 25.6 |
| *Cyanopica cyanus* | 13 | 23 | 7 | 0 | 0 | 0 | 0 | 0 | 20.47 |
| *Dacelo novaeguineae* | 19 | 130 | 8 | 2 | 0 | 0 | 0 | 0 | 37.2 |
| *Delichon urbica* | 14 | 54 | 7 | 0 | 0 | 0 | 0 | 0 | 14.5 |
| *Delichon urbica* | 7 | 57 | 2 | 0 | 0 | 0 | 0 | 0 | 23.6 |
| *Dendroica caerulescens* | 31 | 74 | 15 | 3 | 0 | 0 | 0 | 0 | 15 |
| *Dendroica petechia* | 9 | 101 | 19 | 3 | 0 | 0 | 0 | 0 | 34.73 |
| *Euplectes orix* | 38 | 116 | 29 | 0 | 0 | 0 | 0 | 0 | 10.6 |
| *Falco columbarius* | 10 | 18 | 12 | 1 | 0 | 0 | 0 | 0 | 19.46 |
| *Falco columbarius* | 10 | 24 | 7 | 0 | 0 | 0 | 0 | 0 | 17.12 |
| *Falco naumanni* | 15 | 64 | 3 | 0 | 0 | 0 | 0 | 0 | 10.9 |
| *Falco sparverius* | 16 | 59 | 15 | 5 | 0 | 0 | 0 | 0 | 12.2 |
| *Ficedula hypoleuca* | 16 | 146 | 10 | 0 | 0 | 0 | 0 | 0 | 21.7 |
| *Ficedula hypoleuca* | 6 | 191 | 5 | 2 | 1 | 0 | 0 | 0 | 19 |
| *Ficedula hypoleuca* | 6 | 8 | 2 | 0 | 0 | 0 | 0 | 0 | 13.8 |
| *Ficedula hypoleuca* | 6 | 23 | 3 | 0 | 0 | 0 | 0 | 0 | 15.2 |
| *Ficedula hypoleuca* | 7 | 125 | 4 | 0 | 0 | 0 | 0 | 0 | 11.5 |
| *Fregata minor* | 8 | 71 | 19 | 1 | 0 | 0 | 0 | 0 | 14.1 |
| *Fringilla coelebs* | 2 | 17 | 3 | 0 | 0 | 0 | 0 | 0 | 23.81 |
| *Fulmarus glacialis* | 2 | . | . | . | . | . | . | . | . |
| *Gallinula chloropus* | 2 | . | . | . | . | . | . | . | . |
| *Gallinula chloropus* | 2 | . | . | . | . | . | . | . | . |
| *Gavia immer* | 11 | 53 | 2 | 0 | 0 | 0 | 0 | 0 | 17 |
| *Geothlypis trichas* | 9 | 71 | 1 | 0 | 0 | 0 | 0 | 0 | 16 |
| *Grallina cyanoleuca* | 19 | 95 | 5 | 0 | 0 | 0 | 0 | 0 | 11.3 |
| *Grallina cyanoleuca* | 19 | 41 | 3 | 0 | 0 | 0 | 0 | 0 | 11.3 |
| *Haematopus ostralegus* | 2 | 60 | 4 | 0 | 0 | 0 | 0 | 0 | 22 |
| *Hirundo rustica* | 6 | 178 | 10 | 0 | 0 | 0 | 0 | 0 | 20.3 |
| *Hymenolaimus malacorhynchos* | 17 | 13 | 1 | 0 | 0 | 0 | 0 | 0 | 43.6 |
| *Lagopus lagopus* | 53 | . | . | . | . | . | . | . | . |
| *Lanius bucephalus* | 18 | 81 | 6 | 2 | 0 | 0 | 0 | 0 | 46.2 |
| *Lanius minor* | 22 | 120 | 14 | 2 | 0 | 0 | 0 | 0 | 21 |
| *Larus occidentalis* | 2 | 6 | 6 | 1 | 0 | 0 | 0 | 0 | 25.2 |
| *Larus occidentalis* | 2 | 8 | 8 | 0 | 0 | 0 | 0 | 0 | 21.7 |
| *Loxoides bailleui* | 11 | 19 | 1 | 0 | 0 | 0 | 0 | 0 | 17.9 |
| *Loxoides bailleui* | 11 | 13 | 7 | 0 | 0 | 0 | 0 | 0 | 12.2 |
| *Luscinia svecica* | 7 | . | . | . | . | . | . | . | 29.9 |
| *Malurus cyaneus* | 19 | 33 | 9 | 1 | 0 | 0 | 0 | 0 | 36 |
| *Manorina melanophrys* | 9 | 2 | 4 | 7 | 6 | 3 | 0 | 1 | 15.28 |
| *Melanerpes formicivorus* | 20 | 41 | 8 | 1 | 0 | 0 | 0 | 0 | 15 |
| *Merops apiaster* | 21 | 46 | 5 | 0 | 0 | 0 | 0 | 0 | 23.7 |
| *Miliaria calandra* | 2 | 32 | 4 | 0 | 0 | 0 | 0 | 0 | 13.4 |
| *Notiomystis cincta* | 29 | 18 | 4 | 0 | 0 | 0 | 0 | 0 | 19.53 |
| *Notiomystis cincta* | 29 | 20 | 2 | 0 | 0 | 0 | 0 | 0 | 18.63 |
| *Oceanites oceanicus* | 39 | 51 | 12 | 0 | 0 | 0 | 0 | 0 | 38.1 |
| *Oceanodroma leucorhoa* | 8 | 35 | 7 | 0 | 0 | 0 | 0 | 0 | 21.4 |
| *Oenanthe oenanthe* | 2 | 48 | 6 | 0 | 0 | 0 | 0 | 0 | 27.8 |
| *Otus asio* | 3 | 53 | 18 | 5 | 0 | 0 | 0 | 0 | 51.4 |
| *Otus flammeolus* | 54 | 32 | 5 | 0 | 0 | 0 | 0 | 0 | 44 |
| *Panurus biarmicus* | 22 | 129 | 18 | 1 | 0 | 0 | 0 | 0 | 26 |
| *Parus ater* | 16 | 103 | 14 | 1 | 0 | 0 | 0 | 0 | 14.03 |
| *Parus atricapillus* | 9 | 8 | 30 | 6 | 0 | 0 | 0 | 0 | 20.26 |
| *Parus caeruleus* | 23 | . | . | . | . | . | . | . | 20 |
| *Parus caeruleus* | 23 | 740 | 31 | 0 | 0 | 0 | 0 | 0 | 18.2 |
| *Parus caeruleus* | 6 | 44 | 4 | 0 | 0 | 0 | 0 | 0 | 29.4 |
| *Parus cristatus* | 23 | . | . | . | . | . | . | . | 19 |
| *Parus major* | 24 | 455 | 20 | 0 | 0 | 0 | 0 | 0 | 27.9 |
| *Parus major* | 24 | 471 | 9 | 0 | 0 | 0 | 0 | 0 | 33.4 |
| *Parus major* | 6 | 34 | 4 | 0 | 2 | 0 | 0 | 0 | 31.4 |
| *Parus montanus* | 25 | 92 | 12 | 6 | 1 | 0 | 0 | 0 | 19.5 |
| *Passer domesticus* | 10 | 18 | 0 | 0 | 0 | 0 | 0 | 0 | 14.25 |
| *Passer domesticus* | 2 | 9 | 1 | 0 | 0 | 0 | 0 | 0 | 28.8 |
| *Passer domesticus* | 40 | 131 | 9 | 0 | 0 | 0 | 0 | 0 | 12.98 |
| *Passer domesticus* | 41 | 81 | 23 | 4 | 0 | 1 | 0 | 0 | 38.6 |
| *Passer domesticus* | 10 | 366 | 3 | 0 | 0 | 0 | 0 | 0 | 12.88 |
| *Passer domesticus* | 22 | 80 | 9 | 7 | 0 | 0 | 0 | 0 | 29.7 |
| *Passerculus sandwichensis* | 26 | . | . | . | . | . | . | . | 16 |
| *Passerina cyanea* | 3 | 28 | 10 | 3 | 0 | 0 | 0 | 0 | 37.5 |
| *Perisoreus infaustus* | 6 | 20 | 0 | 0 | 0 | 0 | 0 | 0 | 19 |
| *Petroica australis* | 29 | . | . | . | . | . | . | . | 34.9 |
| *Petroica australis* | 29 | 27 | 4 | 0 | 0 | 0 | 0 | 0 | 37 |
| *Petronia petronia* | 42 | . | . | . | . | . | . | . | 23.9 |
| *Phainopepla nitens* | 31 | 28 | 6 | 0 | 0 | 0 | 0 | 0 | 27.8 |
| *Phalacrocorax aristotelis* | 27 | 21 | 2 | 0 | 0 | 0 | 0 | 0 | 16.07 |
| *Phalaropus lobatus* | 3 | 93 | 22 | 14 | 6 | 5 | 0 | 0 | 51.1 |
| *Phalaropus tricolor* | 9 | 33 | 9 | 1 | 0 | 0 | 0 | 0 | 14.2 |
| *Phoebastria irrorata* | 8 | 11 | 1 | 0 | 0 | 0 | 0 | 0 | 15.8 |
| *Phoebastria irrorata* | 8 | 123 | 5 | 0 | 0 | 0 | 0 | 0 | 15.1 |
| *Phylloscopus trochilus* | 7 | 53 | 15 | 5 | 0 | 0 | 0 | 0 | 28.6 |
| *Pica pica* | 2 | 10 | 6 | 1 | 0 | 0 | 0 | 0 | 21 |
| *Picoides borealis* | 28 | 79 | 0 | 0 | 0 | 0 | 0 | 0 | 45.1 |
| *Porphyrio hochstetteri* | 43 | 27 | 0 | 0 | 0 | 0 | 0 | 0 | 12.15 |
| *Porphyrio porphyrio* | 29 | . | . | . | . | . | . | . | . |
| *Progne subis* | 30 | . | . | . | . | . | . | . | 17 |
| *Progne subis* | 30 | 31 | 3 | 0 | 0 | 0 | 0 | 0 | 21.9 |
| *Prunella collaris* | 2 | 55 | 10 | 0 | 0 | 0 | 0 | 0 | 27.9 |
| *Prunella modularis* | 2 | 129 | 2 | 1 | 0 | 0 | 0 | 0 | 23.2 |
| *Psaltriparus minimus* | 35 | . | . | . | . | . | . | . | 19.1 |
| *Puffinus tenuirostris* | 10 | 7 | 0 | 0 | 0 | 0 | 0 | 0 | 15.7 |
| *Pygoscelis adeliae* | 42 | 15 | 3 | 2 | 0 | 0 | 0 | 0 | 18 |
| *Pygoscelis antarctica* | 55 | 70 | 6 | 0 | 0 | 0 | 0 | 0 | 10.6 |
| *Remiz pendulinus* | 22 | 64 | 34 | 17 | 0 | 0 | 0 | 0 | 11.4 |
| *Riparia riparia* | 14 | 124 | 9 | 2 | 1 | 0 | 0 | 0 | 20 |
| *Riparia riparia* | 22 | 78 | 35 | 16 | 2 | 0 | 0 | 0 | 21.2 |
| *Sayornis phoebe* | 9 | 57 | 9 | 1 | 0 | 0 | 0 | 0 | 41.25 |
| *Sericornis frontinalis* | 9 | 112 | 8 | 0 | 0 | 0 | 0 | 0 | 30 |
| *Serinus canaria* | 23 | 43 | 2 | 0 | 0 | 0 | 0 | 0 | 24.1 |
| *Serinus serinus* | 22 | . | . | . | . | . | . | . | . |
| *Serinus serinus* | 22 | 109 | 14 | 1 | 0 | 0 | 0 | 0 | 21 |
| *Setophaga ruticilla* | 31 | 57 | 5 | 0 | 0 | 0 | 0 | 0 | 15.4 |
| *Sialia mexicanus* | 20 | . | . | . | . | . | . | . | . |
| *Sialia sialis* | 9 | 17 | 4 | 0 | 0 | 0 | 0 | 0 | 20.98 |
| *Spheniscus humboldti* | 11 | 40 | 8 | 1 | 0 | 0 | 0 | 0 | 36.4 |
| *Sterna hirundo* | 56 | 19 | 3 | 2 | 0 | 0 | 0 | 0 | 20.5 |
| *Sturnus unicolor* | 44 | 248 | 20 | 2 | 0 | 0 | 0 | 0 | 17.06 |
| *Sturnus vulgaris* | 2 | 47 | 3 | 1 | 0 | 0 | 0 | 0 | 31.6 |
| *Sturnus vulgaris* | 2 | 45 | 5 | 1 | 0 | 0 | 0 | 0 | 30.9 |
| *Sturnus vulgaris* | 32 | 81 | 2 | 1 | 0 | 0 | 0 | 0 | 16 |
| *Tachycineta bicolor* | 9 | 43 | 9 | 1 | 0 | 0 | 0 | 0 | 30.9 |
| *Tachycineta bicolor* | 9 | 32 | 3 | 1 | 0 | 0 | 0 | 0 | 30.9 |
| *Tachycineta bicolor* | 9 | 4 | 4 | 7 | 6 | 7 | 0 | 0 | 50 |
| *Taeniopygia guttata* | 2 | 75 | 4 | 1 | 0 | 0 | 0 | 0 | 22.8 |
| *Tetrao tetrix* | 2 | 45 | 17 | 0 | 0 | 0 | 0 | 0 | 33.6 |
| *Thalassoica antarctica* | 7 | 27 | 11 | 0 | 0 | 0 | 0 | 0 | 30.9 |
| *Thryothorus ludovicianus* | 45 | 68 | 13 | 2 | 1 | 0 | 0 | 0 | 13.3 |
| *Tockus monteiri* | 46 | 109 | 20 | 6 | 0 | 0 | 0 | 0 | 16.62 |
| *Tribonyx mortierii* | 9 | . | . | . | . | . | . | . | . |
| *Troglodytes aedon* | 33 | 710 | 27 | 4 | 1 | 0 | 0 | 0 | 37.5 |
| *Turdoides squamiceps* | 8 | 178 | 8 | 0 | 0 | 0 | 0 | 0 | 64.1 |
| *Turdus grayi* | 30 | 18 | 3 | 0 | 0 | 0 | 0 | 0 | 15.7 |
| *Tyrannus tyrannus* | 47 | 24 | 12 | 1 | 0 | 0 | 0 | 0 | 14 |
| *Tyto alba* | 16 | 105 | 16 | 0 | 0 | 0 | 0 | 0 | 26.8 |
| *Tyto alba* | 16 | 89 | 0 | 0 | 0 | 0 | 0 | 0 | 8.35 |
| *Upupa epops* | 48 | 68 | 21 | 8 | 2 | 0 | 0 | 0 | 19.4 |
| *Uria aalge* | 2 | . | . | . | . | . | . | . | . |
| *Vireo solitarius* | 30 | 36 | 0 | 0 | 0 | 0 | 0 | 0 | 16.4 |
| *Wilsonia citrina* | 11 | . | . | . | . | . | . | . | 14.3 |
| *Wilsonia citrina* | 30 | 41 | 8 | 6 | 0 | 0 | 0 | 0 | 23 |
| *Zonotrichia albicollis* | 49 | . | . | . | . | . | 0 | 0 | . |
| *Zosterops lateralis* | 50 | 54 | 0 | 0 | 0 | 0 | 0 | 0 | 13.7 |

| Species | No. offspring used for mutation estimate | Total number of offspring | Estimated mutation rate | Mutation rate in publication | Extra-pair paternity (%) | Genetic marker | Genetic marker is a minisatellite | Study included in Amos [1] | Reference |
| --- | --- | --- | --- | --- | --- | --- | --- | --- | --- |
| *Acrocephalus arundinaceus* | 536 | 678 | 0.003827 | . | 3.07 | 33.15 | 1 | 0 |  |
| *Acrocephalus melanopogon* | 18 | 44 | 0.015518 | . | 27.27 | per | 1 | 0 |  |
| *Acrocephalus schoenobaenus* | 143 | 201 | 0.006145 | . | 7.46 | 33.15, 33.6 | 1 | 0 |  |
| *Acrocephalus schoenobaenus* | 117 | 143 | 0.006295 | 0.008000 | 8.39 | 33.15, 33.6 | 1 | 1 | [1] |
| *Acrocephalus vaughani* | 20 | 42 | . | 0.000000 | 0.00 | 33.15 | 1 | 0 |  |
| *Actitis hypoleucos* | 60 | 80 | 0.007077 | 0.006000 | 15.66 | 33.6, 33.15 | 1 | 0 |  |
| *Agelaius phoeniceus* | 176 | 235 | 0.008332 | 0.008000 | 24.68 | M13, M2.5, 18.15 | 1 | 0 |  |
| *Agelaius phoeniceus* | 267 | 403 | . | 0.013910 | 33.75 | M13, per, 33.6 | 1 | 0 |  |
| *Alectura lathami* | 50 | 65 | 0.003817 | 0.003333 | 27.69 | 18.15 | 1 | 0 |  |
| *Anser caerulescens* | 75 | 80 | 0.001699 | . | 5.00 | per, M13 | 1 | 0 |  |
| *Anser rossi* | 81 | 83 | 0.003086 | . | 2.41 | per, M13 | 1 | 0 |  |
| *Anthus spinoletta* | 681 | 1052 | 0.077632 | . | 5.23 | 33.15 | 1 | 0 |  |
| *Aphelocoma coerulescens* | 139 | 139 | 0.005507 | 0.005000 | 0.00 | 33.15, pSP2.5R1 | 1 | 0 |  |
| *Apus apus* | 84 | 88 | 0.013500 | 0.013000 | 4.55 | 33.15 | 1 | 0 |  |
| *Asio otus* | 59 | 59 | 0.002365 | 0.001800 | 0.00 | 33.15 | 1 | 0 |  |
| *Athene noctua* | 53 | 53 | 0.003602 | 0.003500 | 0.00 | (CA)8 | 0 | 0 |  |
| *Branta leucopsis* | 14 | 18 | 0.019380 | 0.013800 | 0.00 | 33.15 | 1 | 0 |  |
| *Branta leucopsis* | 14 | 18 | 0.022624 | 0.006500 | 0.00 | 33.6 | 1 | 0 |  |
| *Branta leucopsis* | 114 | 137 | 0.000820 | 0.000800 | 0.00 | 33.15 | 1 | 0 |  |
| *Buteo galapagoensis* | 19 | 19 | 0.001389 | 0.001200 | 0.00 | 33.15, 33.6 | 1 | 1 | [2] |
| *Calidris maritima* | 81 | 82 | 0.007716 | 0.008000 | 1.22 | per | 1 | 0 |  |
| *Calidris mauri* | 57 | 98 | 0.011513 | . | 5.10 | per | 1 | 0 |  |
| *Calonectris diomedea* | 34 | 34 | 0.007711 | 0.007000 | 0.00 | (GGAT)4 | 0 | 0 |  |
| *Campylorhynchus griseus* | 207 | 222 | 0.004685 | 0.004700 | 2.30 | 33.6, 33.15 | 1 | 0 |  |
| *Campylorhynchus griseus* | 207 | 222 | . | 0.003900 | 2.30 | 33.6, 33.15 | 1 | 0 |  |
| *Campylorhynchus nuchalis* | 68 | 69 | 0.002703 | . | 1.40 | 33.6, 33.15 | 1 | 0 |  |
| *Cardinalis cardinalis* | 32 | 37 | 0.013725 | . | 13.51 | M13 | 1 | 0 |  |
| *Cardinalis cardinalis* | 32 | 37 | 0.010663 | . | 13.51 | per | 1 | 0 |  |
| *Carduelis tristis* | 54 | 70 | 0.012963 | . | 14.29 | 33.15 | 1 | 0 |  |
| *Carpodacus mexicanus* | 73 | 119 | 0.001218 | . | 8.40 | 33.15, per | 1 | 0 |  |
| *Catharacta lonnbergi* | 45 | 45 | . | 0.001400 | 0.00 | 33.15, 33.6 | 1 | 0 |  |
| *Catharacta maccormicki* | 12 | 14 | 0.003104 | . | 7.14 | 33.15 | 1 | 0 |  |
| *Cercomacra tyrannina* | 11 | 15 | 0.023377 | . | 0.00 | 33.15, 33.6 | 1 | 0 |  |
| *Charadrius alexandrinus* | 167 | 229 | 0.018297 | 0.015000 | 1.31 | per | 1 | 0 |  |
| *Charadrius hiaticula* | 50 | 57 | 0.016760 | . | 0.00 | 33.15 | 1 | 0 |  |
| *Charadrius semipalmatus* | 62 | 85 | 0.001182 | 0.001200 | 4.71 | 33.15 | 1 | 0 |  |
| *Coragyps atratus* | 36 | 36 | 0.003840 | 0.004000 | 0.00 | 33.6, 33.15, M13 | 1 | 0 |  |
| *Coragyps atratus* | 36 | 36 | 0.006092 | 0.004000 | 0.00 | 33.6, 33.15, M13 | 1 | 0 |  |
| *Corcorax melanoramphos* | 51 | 51 | 0.007863 | . | 0.00 | per, 33.15 | 1 | 1 | [3] |
| *Corvus monedula* | 74 | 74 | . | 0.004386 | 0.00 | 33.15, 33.6 | 1 | 0 |  |
| *Cyanoliseus patagonus* | 164 | 166 | 0.003335 | 0.003000 | 0.00 | (CA)8 | 0 | 0 |  |
| *Cyanopica cyanus* | 30 | 30 | 0.011399 | . | 0.00 | Jeffreys 33.6 | 1 | 0 |  |
| *Dacelo novaeguineae* | 140 | 140 | 0.002304 | 0.002200 | 0.00 | 33.15, 33.6 | 1 | 1 | [4] |
| *Delichon urbica* | 61 | 72 | 0.007914 | . | 14.52 | 33.15 | 1 | 0 |  |
| *Delichon urbica* | 59 | 73 | 0.001436 | 0.006000 | 19.18 | 33.15 | 1 | 0 |  |
| *Dendroica caerulescens* | 92 | 125 | 0.015217 | 0.012000 | 27.20 | (GGAT)4 | 0 | 0 |  |
| *Dendroica petechia* | 123 | 355 | 0.005852 | 0.004600 | 36.62 | 33.15, per | 1 | 0 |  |
| *Euplectes orix* | 145 | 432 | 0.018868 | 0.019000 | 17.59 | (GGAT)4 | 0 | 0 |  |
| *Falco columbarius* | 31 | 54 | 0.023207 | . | 0.00 | 33.6 | 1 | 0 |  |
| *Falco columbarius* | 31 | 54 | 0.013190 | . | 0.00 | 33.15 | 1 | 0 |  |
| *Falco naumanni* | 67 | 147 | 0.004108 | 0.004100 | 3.45 | (GGAT)4 | 0 | 0 |  |
| *Falco sparverius* | 79 | 89 | 0.025939 | . | 11.24 | (GGAT)4 | 0 | 0 |  |
| *Ficedula hypoleuca* | 156 | 165 | 0.002954 | 0.003000 | 5.45 | CA8 | 0 | 0 |  |
| *Ficedula hypoleuca* | 199 | 223 | 0.003174 | . | 10.76 | 33.15 | 1 | 0 |  |
| *Ficedula hypoleuca* | 10 | 38 | 0.014493 | . | 23.68 | M13 | 1 | 0 |  |
| *Ficedula hypoleuca* | 26 | 38 | 0.007591 | . | 23.68 | 33.15 | 1 | 0 |  |
| *Ficedula hypoleuca* | 129 | 135 | 0.002696 | . | 4.44 | 3'HVR | 1 | 0 |  |
| *Fregata minor* | 91 | 92 | 0.016367 | 0.016400 | 1.09 | 33.15 | 1 | 0 |  |
| *Fringilla coelebs* | 20 | 47 | 0.006300 | 0.007000 | 17.02 | 33.6 | 1 | 0 |  |
| *Fulmarus glacialis* | 19 | 19 | . | 0.017000 | 0.00 | 33.15 | 1 | 0 |  |
| *Gallinula chloropus* | 90 | 90 | . | 0.002500 | 0.00 | 33.15 | 1 | 0 |  |
| *Gallinula chloropus* | 90 | 90 | . | 0.002500 | 0.00 | cGaMSO2 | 1 | 0 |  |
| *Gavia immer* | 55 | 58 | 0.002139 | . | 0.00 | 33.15 | 1 | 0 |  |
| *Geothlypis trichas* | 72 | 153 | 0.000868 | 0.001020 | 20.26 | 33.15, per | 1 | 1 | [5] |
| *Grallina cyanoleuca* | 100 | 103 | 0.004425 | . | 2.91 | per | 1 | 0 |  |
| *Grallina cyanoleuca* | 44 | 103 | 0.006034 | . | 2.91 | per | 1 | 0 |  |
| *Haematopus ostralegus* | 64 | 65 | 0.002841 | . | 1.54 | 33.6 | 1 | 0 |  |
| *Hirundo rustica* | 188 | 261 | 0.002620 | 0.002000 | 27.97 | 33.15 | 1 | 0 |  |
| *Hymenolaimus malacorhynchos* | 14 | 14 | 0.001638 | . | 0.00 | 33.15, 3'HVR | 1 | 0 |  |
| *Lagopus lagopus* | 232 | 256 | . | 0.008200 | 9.38 | per, 33.15, 33.6 | 1 | 0 |  |
| *Lanius bucephalus* | 89 | 99 | 0.002432 | 0.004400 | 10.10 | 33.6, 33.15 | 1 | 0 |  |
| *Lanius minor* | 136 | 136 | 0.006303 | . | 0.00 | (GATA)4 | 0 | 0 |  |
| *Larus occidentalis* | 13 | 33 | 0.024420 | . | 0.00 | 33.15 | 1 | 0 |  |
| *Larus occidentalis* | 16 | 33 | 0.023041 | . | 0.00 | 33.6 | 1 | 0 |  |
| *Loxoides bailleui* | 20 | 20 | 0.002793 | 0.004000 | 0.00 | M13 | 1 | 0 |  |
| *Loxoides bailleui* | 20 | 20 | 0.028689 | 0.014000 | 0.00 | 33.15 | 1 | 0 |  |
| *Luscinia svecica* | 120 | 150 | . | 0.015318 | 20.00 | 33.15 | 1 | 0 |  |
| *Malurus cyaneus* | 43 | 181 | 0.007106 | . | 76.24 | 33.15, per | 1 | 0 |  |
| *Manorina melanophrys* | 23 | 24 | 0.153654 | . | 4.17 | 33.15 | 1 | 0 |  |
| *Melanerpes formicivorus* | 50 | 51 | 0.013333 | . | 0.00 | 33.15 | 1 | 1 | [6] |
| *Merops apiaster* | 51 | 100 | 0.004137 | 0.004100 | 1.00 | 33.15 | 1 | 0 |  |
| *Miliaria calandra* | 36 | 44 | 0.008292 | 0.008264 | 4.55 | 33.6 | 1 | 0 |  |
| *Notiomystis cincta* | 22 | 34 | 0.009310 | 0.007550 | 35.29 | 33.15 | 1 | 0 |  |
| *Notiomystis cincta* | 22 | 34 | 0.004880 | 0.007550 | 35.29 | CA | 1 | 0 |  |
| *Oceanites oceanicus* | 63 | 63 | 0.004999 | 0.005000 | 0.00 | (GGAT)4 | 0 | 0 |  |
| *Oceanodroma leucorhoa* | 42 | 48 | 0.007788 | 0.008000 | 0.00 | 33.15 | 1 | 0 |  |
| *Oenanthe oenanthe* | 54 | 73 | 0.003997 | 0.003861 | 10.96 | 33.15 | 1 | 0 |  |
| *Otus asio* | 76 | 80 | 0.007168 | . | 0.00 | M13, per | 1 | 0 |  |
| *Otus flammeolus* | 37 | 37 | 0.003071 | 0.003100 | 0.00 | 33.15, 33.6 | 1 | 0 |  |
| *Panurus biarmicus* | 148 | 187 | 0.005198 | . | 14.44 | (GATA)4 | 0 | 0 |  |
| *Parus ater* | 118 | 158 | 0.009665 | 0.006300 | 25.32 | (CA)8 | 0 | 0 |  |
| *Parus atricapillus* | 44 | 53 | 0.047115 | . | 16.98 | per, 33.15 | 1 | 0 |  |
| *Parus caeruleus* | 265 | 314 | . | 0.002000 | 10.51 | 33.15 | 1 | 0 |  |
| *Parus caeruleus* | 771 | 864 | 0.002209 | . | 10.76 | 33.15 | 1 | 0 |  |
| *Parus caeruleus* | 48 | 51 | 0.002834 | 0.003000 | 5.88 | M13, 33.15 | 1 | 0 |  |
| *Parus cristatus* | 106 | 121 | . | 0.009000 | 12.40 | 33.15 | 1 | 0 |  |
| *Parus major* | 475 | 516 | 0.001509 | 0.001500 | 3.49 | 33.15 | 1 | 0 |  |
| *Parus major* | 480 | 516 | 0.000561 | 0.000569 | 3.49 | 33.6 | 1 | 0 |  |
| *Parus major* | 40 | 47 | 0.007962 | 0.008000 | 24.89 | M13, 33.15 | 1 | 0 |  |
| *Parus montanus* | 111 | 112 | 0.012474 | . | 0.89 | (GTG)5 | 0 | 0 |  |
| *Passer domesticus* | 18 | 19 | 0.000000 | 0.000000 | 5.26 | 33.6 | 1 | 0 |  |
| *Passer domesticus* | 10 | 11 | 0.003472 | 0.003500 | 9.09 | 33.6, 33.15 | 1 | 0 |  |
| *Passer domesticus* | 139 | 171 | 0.004988 | 0.013500 | 7.02 | (GGAT)4 | 0 | 0 |  |
| *Passer domesticus* | 109 | 136 | 0.008319 | 0.008000 | 19.85 | M13, 33.6 | 1 | 0 |  |
| *Passer domesticus* | 369 | 420 | 0.000631 | 0.001300 | 12.14 | 36.6 | 1 | 0 |  |
| *Passer domesticus* | 96 | 123 | 0.008067 | . | 19.51 | per | 1 | 0 |  |
| *Passerculus sandwichensis* | 116 | 92 | . | 0.024375 | 33.70 | 33.15 | 1 | 0 |  |
| *Passerina cyanea* | 41 | 63 | 0.010407 | 0.011000 | 34.92 | 33.15, 33.6, M13 | 1 | 0 |  |
| *Perisoreus infaustus* | 20 | 20 | 0.000000 | 0.000000 | 0.00 | 33.15 | 1 | 1 | [7] |
| *Petroica australis* | 29 | 29 | . | 0.000772 | 0.00 | 33.15, 33.6 | 1 | 0 |  |
| *Petroica australis* | 32 | 32 | 0.003378 | 0.003273 | 0.00 | 33.15, 33.6 | 1 | 0 |  |
| *Petronia petronia* | 123 | 181 | . | 0.014000 | 32.04 | 33.15 | 1 | 1 | [8] |
| *Phainopepla nitens* | 34 | 48 | 0.006348 | 0.004494 | 0.00 | 33.15 | 1 | 0 |  |
| *Phalacrocorax aristotelis* | 23 | 28 | 0.005411 | . | 17.86 | 33.15 | 1 | 0 |  |
| *Phalaropus lobatus* | 140 | 232 | 0.012301 | . | 1.72 | 33.15, M13 | 1 | 0 |  |
| *Phalaropus tricolor* | 43 | 51 | 0.018015 | 0.021000 | 0.00 | 33.15 | 1 | 0 |  |
| *Phoebastria irrorata* | 12 | 16 | 0.005274 | . | 25.00 | 33.15 | 1 | 0 |  |
| *Phoebastria irrorata* | 128 | 154 | 0.002587 | . | 16.88 | 33.15 | 1 | 0 |  |
| *Phylloscopus trochilus* | 73 | 109 | 0.011974 | . | 33.03 | per | 1 | 0 |  |
| *Pica pica* | 17 | 17 | 0.022409 | . | 3.13 | 33.15 | 1 | 0 |  |
| *Picoides borealis* | 79 | 80 | 0.000000 | 0.000000 | 1.25 | per | 1 | 0 |  |
| *Porphyrio hochstetteri* | 27 | 27 | 0.000000 | 0.000000 | 0.00 | 33.6, pV47-2 | 1 | 1 | [9] |
| *Porphyrio porphyrio* | 74 | 74 | . | 0.001380 | 0.00 | pV47-2, 3'HVR, per | 1 | 0 |  |
| *Progne subis* | 116 | 138 | . | 0.013529 | 18.84 | 33.6 | 1 | 0 |  |
| *Progne subis* | 34 | 52 | 0.004029 | . | 34.62 | 33.6 | 1 | 0 |  |
| *Prunella collaris* | 65 | 110 | 0.005514 | 0.005525 | 0.00 | 33.15, 33.6 | 1 | 0 |  |
| *Prunella modularis* | 132 | 133 | 0.001306 | 0.000100 | 0.80 | 33.15 | 1 | 0 |  |
| *Psaltriparus minimus* | 50 | 50 | . | 0.002000 | 0.00 | per, 33.15, 33.6 | 1 | 0 |  |
| *Puffinus tenuirostris* | 7 | 22 | 0.000000 | 0.000000 | 10.80 | 33.6 | 1 | 0 |  |
| *Pygoscelis adeliae* | 20 | 22 | 0.019444 | 0.013900 | 9.09 | 33.15 | 1 | 1 | [10] |
| *Pygoscelis antarctica* | 76 | 76 | 0.007448 | 0.007400 | 0.00 | (GGAT)4 | 0 | 0 |  |
| *Remiz pendulinus* | 115 | 201 | 0.050125 | . | 6.97 | (GATA)4 | 0 | 0 |  |
| *Riparia riparia* | 136 | 167 | 0.005882 | . | 14.37 | 33.6 | 1 | 0 |  |
| *Riparia riparia* | 131 | 190 | 0.026285 | . | 18.95 | per | 1 | 0 |  |
| *Sayornis phoebe* | 67 | 76 | 0.003980 | . | 11.84 | 33.15, per | 1 | 0 |  |
| *Sericornis frontinalis* | 120 | 137 | 0.002222 | 0.005600 | 12.41 | per, 33.15 | 1 | 0 |  |
| *Serinus canaria* | 45 | 45 | 0.001844 | 0.002000 | 0.00 | 33.15 | 1 | 0 |  |
| *Serinus serinus* | 61 | 61 | . | 0.001429 | 0.00 | (GATA)4 | 0 | 1 | [11] |
| *Serinus serinus* | 124 | 139 | 0.006144 | . | 9.40 | (GATA)4 | 0 | 0 |  |
| *Setophaga ruticilla* | 62 | 108 | 0.005237 | . | 39.81 | (GGAT)4 | 0 | 0 |  |
| *Sialia mexicanus* | 127 | 207 | . | 0.012000 | 18.36 | 33.15, 33.6 | 1 | 0 |  |
| *Sialia sialis* | 21 | 83 | 0.009079 | . | 8.43 | 33.15 | 1 | 0 |  |
| *Spheniscus humboldti* | 49 | 49 | 0.005607 | 0.014500 | 0.00 | 33.15, 33.6 | 1 | 0 |  |
| *Sterna hirundo* | 24 | 29 | 0.014228 | 0.012700 | 0.00 | 33.15 | 1 | 0 |  |
| *Sturnus unicolor* | 270 | 334 | 0.005210 | 0.005000 | 15.87 | 33.15 | 1 | 0 |  |
| *Sturnus vulgaris* | 51 | 62 | 0.003103 | . | 9.68 | 33.6 | 1 | 0 |  |
| *Sturnus vulgaris* | 51 | 62 | 0.004442 | . | 9.68 | 33.15 | 1 | 0 |  |
| *Sturnus vulgaris* | 84 | 92 | 0.002976 | . | 8.70 | 33.15 | 1 | 0 |  |
| *Tachycineta bicolor* | 53 | 86 | 0.006717 | . | 38.37 | per | 1 | 0 |  |
| *Tachycineta bicolor* | 36 | 95 | 0.004495 | . | 62.11 | 33.15, per | 1 | 0 |  |
| *Tachycineta bicolor* | 28 | 111 | 0.045714 | . | 68.47 | per, 33.15 | 1 | 0 |  |
| *Taeniopygia guttata* | 80 | 92 | 0.003289 | . | 2.17 | 33.6, 33.15 | 1 | 0 |  |
| *Tetrao tetrix* | 62 | 66 | 0.008161 | . | 0.00 | 33.6 | 1 | 1 | [12] |
| *Thalassoica antarctica* | 38 | 41 | 0.009368 | . | 7.32 | per | 1 | 0 |  |
| *Thryothorus ludovicianus* | 84 | 84 | 0.017902 | 0.019000 | 0.00 | 33.15 | 1 | 0 |  |
| *Tockus monteiri* | 135 | 135 | 0.014262 | 0.014000 | 0.00 | 33.15 | 1 | 0 |  |
| *Tribonyx mortierii* | 26 | 26 | . | 0.018000 | 0.00 | per | 1 | 0 |  |
| *Troglodytes aedon* | 742 | 790 | 0.001366 | 0.001400 | 8.35 | M13, per | 1 | 0 |  |
| *Turdoides squamiceps* | 186 | 186 | 0.000671 | 0.000700 | 0.00 | 33.15, 33.6 | 1 | 0 |  |
| *Turdus grayi* | 21 | 37 | 0.009099 | . | 37.84 | 33.15 | 1 | 0 |  |
| *Tyrannus tyrannus* | 37 | 64 | 0.027027 | 0.030000 | 42.19 | 33.15 | 1 | 1 | [13] |
| *Tyto alba* | 121 | 122 | 0.004934 | . | 0.82 | (GGAT)4 | 0 | 1 | [14] |
| *Tyto alba* | 89 | 89 | 0.000000 | . | 0.00 | (GGAT)4 | 0 | 1 |  |
| *Upupa epops* | 99 | 108 | 0.022389 | . | 8.33 | 33.15 | 1 | 0 |  |
| *Uria aalge* | 71 | 77 | . | 0.019000 | 7.79 | per | 1 | 1 | [15] |
| *Vireo solitarius* | 36 | 37 | 0.000000 | . | 2.70 | 33.15 | 1 | 0 |  |
| *Wilsonia citrina* | 261 | 356 | . | 0.002400 | 26.69 | 33.15 | 1 | 0 |  |
| *Wilsonia citrina* | 55 | 78 | 0.015810 | . | 29.49 | 33.15, 33.6 | 1 | 0 |  |
| *Zonotrichia albicollis* | 71 | 89 | . | 0.013000 | 17.98 | per, M13 | 1 | 0 |  |
| *Zosterops lateralis* | 54 | 122 | 0.000000 | 0.000000 | 0.00 | 33.6 | 1 | 0 |  |

**References**

[1] Buchanan KL, Catchpole CK: Extra-pair paternity in the socially monogamous sedge warbler *Acrocephalus schoenobaenus* as revealed by multilocus DNA fingerprinting. *Ibis* 2000, 142:12-20

[2] Faaborg J, Parker PG, Delay L, De Vries T, Bednarz JC, Paz SM, Naranjo J, Waite TA: Confirmation of cooperative polyandry in the Galapagos hawk (*Buteo galapagoensis*). *Behav Ecol Sociobiol* 1995, 36:83-90

[3] Heinsohn R, Dunn P, Legge S, Double M: Coalitions of relatives and reproductive skew in cooperatively breeding white-winged choughs. *Proc R Soc Lond B* 2000, 267:243-249

[4] Legge S, Cockburn A: Social and mating systems of cooperatively breeding laughing kookaburras (*Dacelo novaeguineae*). *Behav Ecol Sociobiol* 2000, 47:220-229

[5] Peterson KA, Thusius KJ, Whittingham LA, Dunn PO: Allocation of male parental care in relation to paternity within and among broods of the common yellowthroat (*Geothlypis trichas*). *Ethology* 2001, 107:573-586

[6] Dickinson J, Haydock J, Koenig W, Stanback M, Pitelka F: Genetic monogamy in single-male groups of acorn woodpeckers, *Melanerpes formicivorus*. *Mol Ecol* 1995, 4:765-769

[7] Ekman J, Sklepkovych B, Tegelström H: Offspring retention in the Siberian jay (*Perisoreus infaustus*): the prolonged brood care hypothesis. *Behav Ecol* 1994, 5:245-253

[8] Pilastro A, Griggio M, Biddau L, Mingozzi T: Extrapair paternity as a cost of polygyny in the rock sparrow: behavioural and genetic evidence of a ‘trade-off’ hypothesis. *Anim Behav* 2002, 63:967-974

[9] Lettink M, Jamieson IG, Millar CD, Lambert DM: Mating system and genetic variation in the endangered New Zealand takahe. *Cons Genet* 2002, 3:427-434

[10] Mota PG, Hoi-Leitner M: Intense extrapair behaviour in a semicolonial passerine does not result in extrapair fertilizations. *Anim Behav* 2003, 66:1019-1026

[12] Alatalo RV, Burke T, Dann J, Hanotte O, Höglund J, Lundberg A, Moss R, Rintamäki P: Paternity, copulation disturbance and female choice in lekking black grouse. *Anim Behav* 1996, 52:861-873

[13] Rowe DL, Murphy MT, Fleischer RC, Wolf PG 2001. High frequency of extra-pair paternity in eastern kingbirds. *Condor* 2001, 103:845-851

[14] Roulin A, Muller W, Sasvari L, Dijkstra C, Ducrest AL, Riols C, Wink M, Lubjuhn T: Extra-pair paternity, testes size and testosterone level in relation to colour polymorphism in the barn owl *Tyto alba*. *J Avian Biol* 2004, 35:492-500

[15] Birkhead TR, Hatchwell BJ, Lindner R, Blomqvist D, Pellatt EJ, Griffith R, Lifjeld JT: Extra-pair paternity in the Common Murre. *Condor* 2001, 103:158-162
